# Screening novel stress granule regulators from a natural compound library

**DOI:** 10.1007/s13238-017-0430-6

**Published:** 2017-07-10

**Authors:** Li-Dan Hu, Xiang-Jun Chen, Xiao-Yan Liao, Yong-Bin Yan

**Affiliations:** State Key Laboratory of Membrane Biology, School of Life Sciences, Tsinghua University, Beijing, 100084 China

**Dear Editor**,

Modulation of mRNA transportation, localization, translational efficiency, and degradation plays an important role in the regulation of gene expression. In eukaryotic cells, translationally repressed mRNAs may be recruited into distinct intracellular foci termed as RNA granules, which are microscopically visible non-membrane-bound organelles composed of messenger ribonucleoproteins (mRNPs) (Anderson and Kedersha, 2009). Stress granule (SG) and processing body (P body) are two evolutionarily conserved cytoplasmic RNA granules in somatic cells (Anderson and Kedersha, 2006). Assembly of SGs can be stimulated by various stresses and SGs will be disassembled after the stress is removed. By shifting the equilibrium between stalled and translating mRNAs, SG formation can modulate stress response of the cells (Protter and Parker, 2016). It is increasingly recognized that aberrant SG assembly/disassembly may affect cell survival and human diseases thereafter (Anderson et al., 2015; Li et al., 2013; Mahboubi and Stochaj, 2017).

SG assembly and disassembly can be influenced by many factors including various endogenous and extracellular stressors, eIF2α phosphorylation and overexpression of SG promoting proteins (Mahboubi and Stochaj, 2017; Panas et al., 2016; Protter and Parker, 2016). Particularly, several RNA-binding proteins such as TIA-1, TIAR, G3BP, CPEB1, and TTP can initiate SG formation and therefore overexpression of these proteins will promote SG formation even under non-stressed conditions (Kedersha et al., 1999; Stoecklin et al., 2004; Tourriere et al., 2003; Wilczynska et al., 2005). Chemical compounds can also modulate SG formation by affecting translation, proteasome activity or endogenous stressors (Mahboubi and Stochaj, 2017). It is worth noting that the chemical compounds identified thus far affect SG assembly/disassembly indirectly. Meanwhile, the action of these compounds may introduce complicated cellular responses and thereby it is difficult to study the role of SGs in specific cellular events. It has been reported that formation of several cellular bodies including SG, P body, and aggresome involves interactions between low-complexity sequences (Kato et al., 2012; Patel et al., 2015; Reijns et al., 2008). This suggests that these microscopic visible membraneless organelles may share some general rules in assembly though they have dissimilar components and morphology. An interesting question is whether there exist specific SG modulators. To address this problem, we screened a compound library obtained from Chinese traditional medical plants by a novel screening strategy (Fig. 1A).

**Figure 1:**
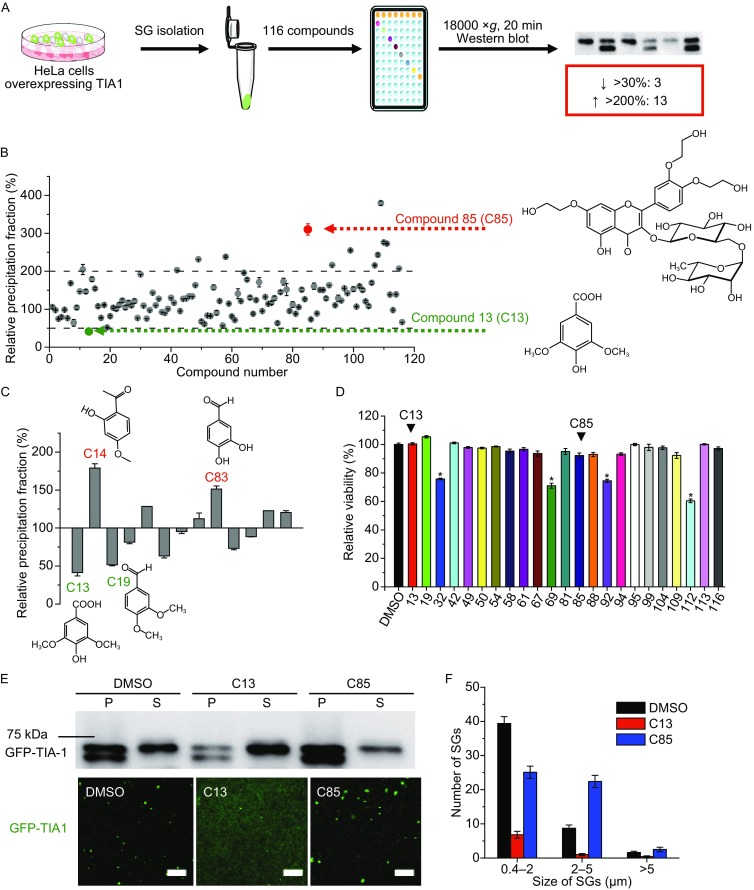
**Screening SG-specific modulators using isolated SG cores from a Chinese traditional plant-derived compound library containing 116 compounds**. (A) A schematic strategy for screening SG-specific chemical compounds. SG cores isolated from cells overexpressing GFP-TIA-1 were divided into small aliquots and treated with compounds from the library. Western blot analysis was used to determine the SG modulators. (B) Quantitative results of the effects of the 80 μmol/L compounds on SG assembly/disassembly *in vitro*. The data were calculated from the ratio of GFP-TIA-1 in the precipitation fraction to that in the supernatant fraction and normalized by taking the control data as 100%. The data of two highly nontoxic compounds are highlighted in red and green and the structures of these two compounds are also shown. The data are presented as mean ± S.E.M. calculated from three independent experiments. (C) Effects of benzene derivatives on SG stability. The structures show four highest activity compounds. (D) Effects of selected compounds with high activity on cell viability. The data are shown as mean ± S.E.M. (*n* = 3). *P < 0.05. (E) Upper panel: representative Western blot analysis of effects of C13 and C85 on isolated SG cores detected by the amounts of GFP-TIA-1 probed by GFP antibody. P and S are the precipitation and supernatant fraction, respectively. Lower panel: representative confocal microscope images of isolated SGs treated with 0.8% DMSO, C13, and C85 in 0.8% DMSO. Scare bar, 20 μm. (F) Size distributions of isolated SGs. The number of small (0.4–2 μm), medium (2–5 μm), and large SGs (>5 μm) were calculated from 10 randomly selected viewing fields

To identify SG specific modulators, SG cores were isolated according to the well-established procedures (Jain et al., 2016) from the TIA-1 overexpressing HeLa cells. These purified SG cores were divided into small aliquots and treated with compounds from a library containing 116 natural compounds isolated from 58 kinds of Chinese traditional medical plants (Fig. 1A). The library covers chemical compounds belonging to alkaloids, glycosides, ketones, flavonoids, phenylpropanoids, phenols, quinones, terpenoids, and steroids. After treatment, the effect of these compounds was determined by the dissociation of GFP-TIA-1 from SGs evaluated by the ratio of precipitation to supernatant fraction. Our results showed that 23 compounds facilitated SG dissociation, while 60 promoted SG assembly (Fig. 1B). Among them, 3 compounds could successfully decrease over 30% of the precipitation fraction, while 13 compounds increased the precipitation fraction above 2-fold. The library contains 13 benzene derivatives. A preliminary analysis suggested that two methoxy groups might be required for the SG dissociation ability (Fig. 1C). Further research using a larger library of benzene derivatives is needed to elucidate the structure-activity relationship. Among the effective compounds, most of them did not affect cell viability, while 4 of them showed significant cytotoxicity at a concentration of 10 μmol/L (Fig. 1D).

We selected two highly effective non-cytotoxic compounds (Fig. 1B), C13 (syringic acid) and C85 (troxerutin), for further investigations. Western blot analysis indicated that C13 and C85 had opposite functions on SG stability (Fig. 1E). Consistently, confocal microscopy of the isolated SG cores treated with C13 showed a disperse GFP fluorescence pattern, which was caused by the re-dissolution of GFP-TIA-1 from SGs. Those treated with C85 had more larger SGs with size above 2 μm when compared with the control (Fig. 1F), implying that C85 could stabilize SGs and perturb the equilibrium between reversible SG assembly and disassembly. C13 and C85 were also very effective for SGs induced by TIA-1 overexpression or arsenite treatment in the HeLa cells (Fig. 2A). Quantitative analysis indicated that the percentage of cells with SGs induced by TIA-1 was significantly reduced by C13 and promoted by C85 in a concentration-dependent manner (Fig. 2B). As for SGs induced by arsenite treatment, C13 decreased both the number and relative mean fluorescence intensity of SGs, while C85 had the opposite effect (Fig. 2C). The addition of C13 or C85 did not affect the formation of protein aggregates formed by a disease-causing mutant αB-crystallin R120G visualized by the fused GFP or P bodies detected by a marker protein EDC4 (Fig. S1) or DCP1a (data not shown). This suggested that C13 and C85 were more likely to be specific modulators of SG formation but not the other types of cytoplasmic granules.

**Figure 2:**
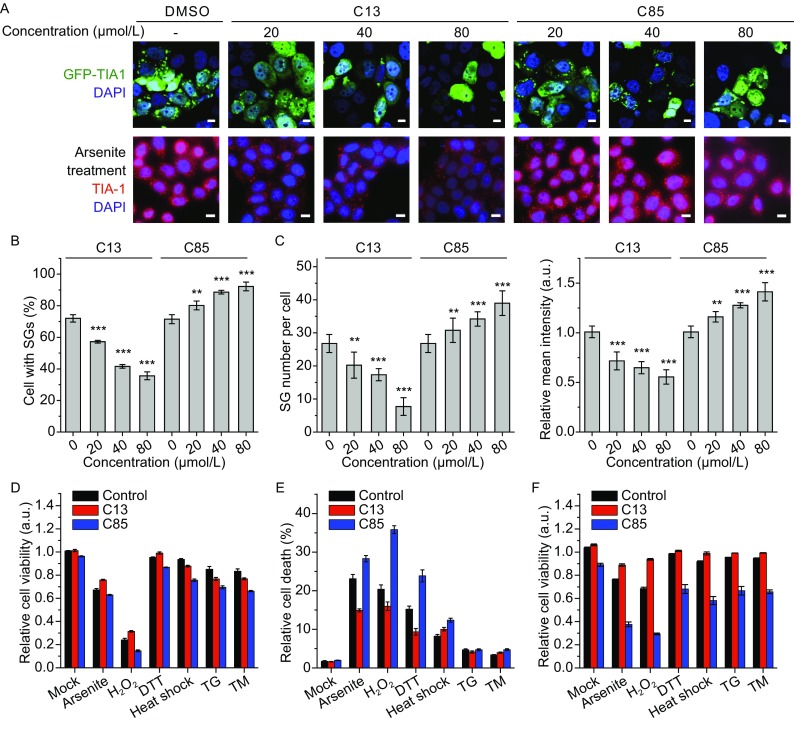
**Verification of the action of C13 and C85 on SGs in HeLa cells**. (A) Representative confocal microscope images showing the effects of various concentrations of C13 and C85 on SGs in HeLa cells induced by overexpression of GFP-TIA-1 or 0.5 mmol/L arsenite for 30 min. Scare bar, 10 μm. Treatment of C13 or C85 did not affect the transient transfection efficiency or expression level of TIA-1 as evaluated by fluorescence mean intensity and Western-blot analysis (data not shown). (B) Quantitative analysis of the actions of C13 and C85 on GFP-TIA-1 overexpression-induced SGs in HeLa cells. The percentages of cells with SGs were calculated from 10 randomly selected viewing fields. (C) Quantitative analysis of the effects of C13 and C85 on the number of SGs per cell and mean fluorescence intensity. SGs were induced by 0.5 mmol/L arsenite for 30 min. **P < 0.01, ***P < 0.001. (D) Effect of the two compounds on cell survival when the HeLa cells were treated by various stressors. Cell viability was measured immediately after the stress treatment to reflect the sensitivity of stress response. (E) Cell death determined by flow cytometry analysis for samples double stained using annexin V and propidium iodide. (F) Effect of the two compounds on the recovery of the HeLa cells from stressed conditions. The cell viability was measured by refreshing the culture medium to remove the stressors and cultivated for 2 h. All cell viability data are presented as mean ± S.E.M. (*n* = 3)

SGs can be induced by many stressors and the compositions may differ for SG induced by different stresses. Both compounds were also effective for SGs induced by H_2_O_2_, heat shock, DTT, TG and TM (Fig. S2A), suggesting that the compounds had a general effect on SG assembly/disassembly in the cells though they were screened using the isolated SG cores induced by TIA-1 overexpression. Quantitative analysis (Fig. S2B) indicated that C13 could decrease the percentage of cells containing SGs when the cells were treated with effective SG inducers such as arsenite, H_2_O_2_, and DTT. For mild SG-inducing conditions including heat shock, TG and TM treatments, C85 enhanced SG formation though C85 could not induce SG formation under normal conditions by itself (data not shown).

Previous functional studies of SGs mainly performed using exogenous stressors or overexpressing/knocking down SG components. It is worth noting that these stressors/proteins may have pleiotropic cellular effects although it is clear that they are involved in SG formation (Mahboubi and Stochaj, 2017; Panas et al., 2016). Both C13 and C85 did not have cytotoxicity (Fig. 1D) and did not induce SG formation under non-stressed conditions (data not shown). Cell viability was further studied under conditions in the presence of stressors. Similar cell survival results were observed in HeLa and HEK 293A cells, and the data of HeLa cells are shown in Figs. 2D–F and S3. Time-course study was performed for cells transfected with GFP-C3 or GFP-TIA-1. The culture medium was not refreshed during cultivation and thereby cells will subject to starvation after 12 h cultivation. Compared to the control group, C13 greatly facilitated cell survival but C85 decreased cell viability after 8 h cultivation (Fig. S3A). Pre-treatment of the cells with the two compounds had dissimilar effects on cellular stress response to arsenite, H_2_O_2_, DTT, heat shock, TG and TM treatments (Fig. 2D and 2E). For all stressors, the SG-promoting compound C85 impaired cell survival and induce cell death. The SG-dissociating compound C13 slightly decreased cell viability for heat shock, TG and TM treatments, while enhanced cell survival and prevented cell death for arsenite, H_2_O_2_, and DTT treatments. The effects of both compounds showed a stressor dose dependency for arsenite and H_2_O_2_ treatments (Fig. S3B). It seems that SG inhibition by C13 affected cell survival differentially for various stresses, which might be caused by the dissimilar compositions of SGs induced by different stressors (Panas et al., 2016) and the severity of the stresses. When the stressors were removed by refreshing the culture medium of the cells, C85 showed ever greater impairments on cell viability, while C13 was beneficial to the cells recovered from all stressed conditions (Fig. 2F). The unappreciated effect of C85 on cell survival was stressor treating-time dependent, while C13 had similar protecting effect for the tested conditions (Fig. S3C).

Formation of SGs has been proposed to facilitate stress response of the eukaryotic cells (Mahboubi and Stochaj, 2017; Protter and Parker, 2016). Although C85 was not cytotoxic under normal cultivating conditions, cells treated with C85 showed hypersensitive to various stressors. The SG-promoting compound C85 was deleterious to not only cell survival under stressed conditions but also recovery after stressors were removed. The extraordinarily enhanced SG formation by C85 might recruit essential stress-fighting mRNAs/proteins into SGs and thereby impair the cellular machines required for survival and recovery. The SG-dissociating compound C13 had complicated effect on cell stress response. Compared with the control group, cells treated with C13 were more resistant to arsenite, H_2_O_2_ and DTT, but was more sensitive to heat shock, TG, and TM. This implied that a proper equilibrium between SG assembly/disassembly was required for cellular stress response. Nonetheless, C13 facilitated the cells recovered from the stressed conditions, suggesting that a faster dissociation of SGs was beneficial to the survived cells to return to their normal states.

In conclusion, herein we developed a simple and effective screening strategy to identify chemical compounds modulating SG assembly/disassembly. A number of natural aromatic compounds had been identified to have the potency to modulate SG formation. The action of two highly effective and nontoxic compounds, C13 (syringic acid) and C85 (troxerutin), were verified for various subtypes of SGs induced by dissimilar stressors. More importantly, both compounds did not affect the formation of other types of cytoplasmic protein aggregates or RNA granules, suggesting that these two compounds were highly SG-specific and could be used to modulate SG formation in the cells without modifications of the other types of cellular bodies. A preliminary functional study indicated that the SG-promoting compound C85 impaired both cellular stress response and recovery from stressed conditions. The SG-dissociating compound C13 had complicated effect on stress response but facilitate recovery after the cells were released from stressors. Our results suggested that there do have SG-specific modulators although the microscopic visible membraneless organelles share some common assembly/disassembly mechanism. Screening using a larger library combined with structural design will provide more SG-specific modulators with higher efficiency. Meanwhile, herein we only performed a preliminary functional study of SGs using these modulators. Further research is needed to elucidate the mechanism of these compounds and their cellular consequence by modulating SG assembly/disassembly.

## FOOTNOTES

This study was supported by funds from the China Postdoctoral Science Foundation (No. 158358) and State Key Laboratory of Membrane Biology (to Y.-B. Yan). Dr. X.-J. Chen was supported by the Excellent Postdoctoral fellowship from the Tsinghua-Peking Joint Center for Life Sciences.

Li-Dan Hu, Xiang-Jun Chen, Xiao-Yan Liao and Yong-Bin Yan declare no conflict of interest. This article does not contain any studies with human or animal subjects performed by the any of the authors.

YBY conceived and designed the experiments. LDH, XJC, and XYL performed the experiments. LDH, XJC, and YBY analyzed the data. LDH and YBY wrote the paper.

## Electronic supplementary material

Below is the link to the electronic supplementary material.

Supplementary material 1 (PDF 248 kb)
