# Supplementary material for: Screening novel stress granule regulators from a natural compound library
Source: Protein Cell. 2017 Jul 10;8(8):618–22. doi: 10.1007/s13238-017-0430-6 (PMC5546934; doi:10.1007/s13238-017-0430-6)
Supplement: Supplementary file 1 — Supplementary material 1 (PDF 248 kb) [file 13238_2017_430_MOESM1_ESM.pdf]

Supplementary Materials

Screening novel stress granule regulators from a natural compound library

Li-Dan Hu, Xiang-Jun Chen, Xiao-Yan Liao, and Yong-Bin Yan*

State Key Laboratory of Membrane Biology, School of Life Sciences, Tsinghua University, Beijing 100084, China

*** To whom all correspondence should be addressed:**

Dr. Yong-Bin Yan, School of Life Sciences, Tsinghua University, Beijing 100084, PR China, Tel: +86-10-62783477, Fax: +86-10-62772245, E-mail: ybyan@tsinghua.edu.cn

Running Title: **Stress granule formation modulated by natural compounds**

Keywords: plant-derived organic compounds; stress granule; stress response; syringic acid; troxerutin

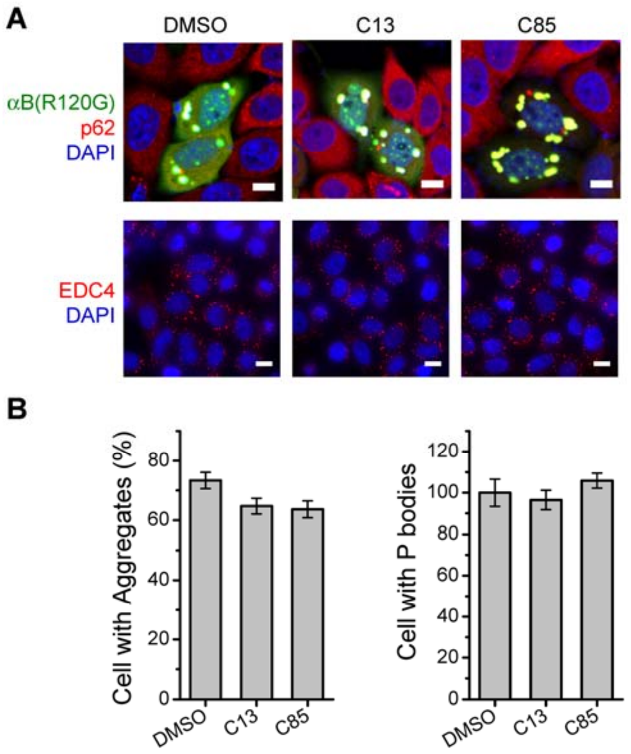

Supplementary Fig. S1. C13 and C85 did not affect formation of intracellular protein aggregates and P-bodies. (A) Representative confocal microscope images showing that C13 and C85 did not affect protein aggregates formed by a disease-causing mutant αB-crystallin R120G (top panel) and P bodies probed by EDC4 antibody (bottom panel). (B) Quantitative analysis of the effects of C13 and C85 on αB-crystallin R120G aggregates (left) and P bodies (right).

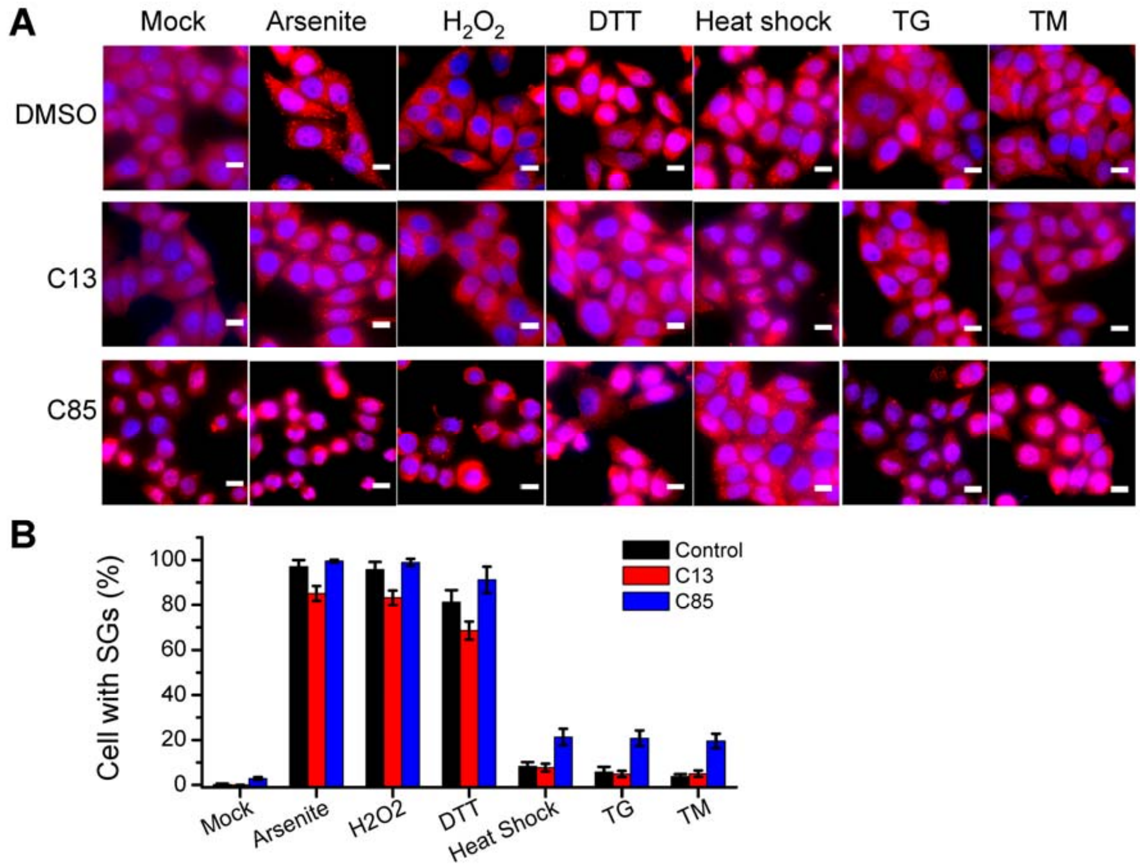

Supplementary Fig. S2. Effect of C13 and C85 on SG formation under various stressed conditions. (A) Representative confocal microscope images showing the effects of C13 and C85 on SGs in HeLa cells induced by various stressors. SGs were visualized by TIA-1 antibody (red). Nuclei were stained by DAPI (blue). Scare bar, 10 μm. (B) Quantitative analysis of the effects of C13 and C85 on the percentages of cells containing SGs. The data were calculated from 10 randomly selected viewing fields.

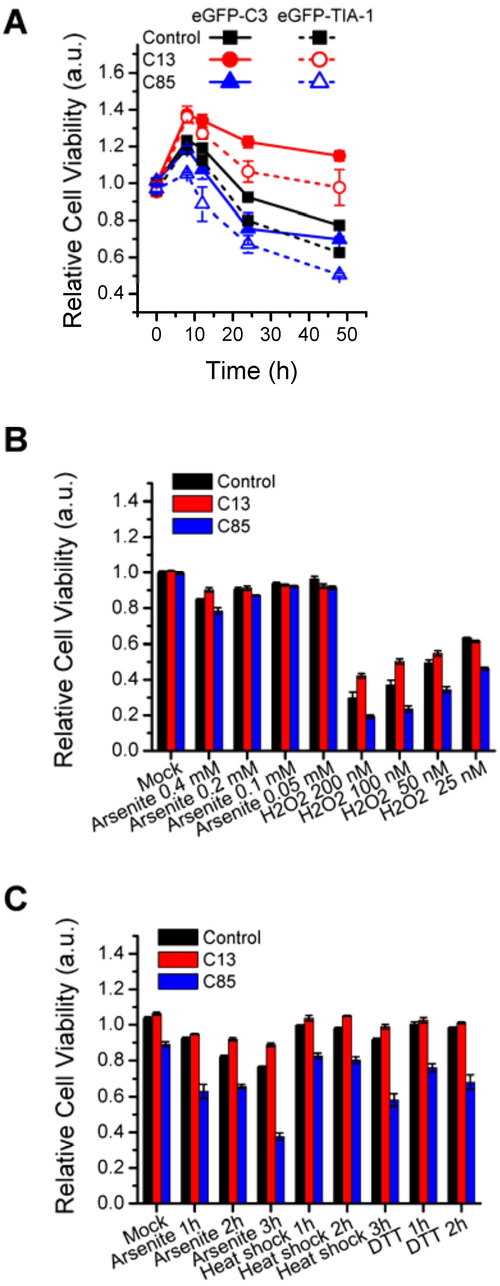

Supplementary Fig. S3. Effect of C13 and C85 on cell viability under various stressed conditions. (A) Time-course study of cell viability in the presence of 80 μM C13 and C85. The cells were transfected with GFP-C3 (control) or GFP-TIA-1 and cultivated without refreshing the culture medium. (B) The action of C13 and C85 was dependent on the concentration of stressors, arsenite and H_2O_2. (C) Effect of treating time on the action of C13 and C85 for cells treated with arsenite and heat shock. All cell viability data are presented as mean ± S.E. (*n*=3).

Materials and Methods

Materials

Dimethyl sulfoxide (DMSO), paraformaldehyde (PFA), Triton X-100, Nonidet P40 (NP-40), phenylmethanesulfonyl fluoride (PMSF), heparin, protease inhibitor cocktail, KCl, $MgCl_2$, thapsigargin (TG) or tunicamycin (TM), DTT and EDTA were purchased from Sigma-Aldrich. TIA-1 rabbit monoclonal antibody, DCP1a rabbit polyclonal antibody and EDC4 rabbit polyclonal antibody were from Abcam. Mouse monoclonal antibody against GFP was obtained from Bioworld Technology. TIA-1(C-20) goat polyclonal antibody was purchased from Santa Cruz Biotechnology. Donkey anti-rabbit Alexa Fluor 549, Donkey anti-goat Alexa Fluor 594, HRP affinipure goat anti-rabbit IgG(H+L) were from EarthOx. DAPI Fluoromount-G was from Southern Biotech. Plasmid Maxipre kit was purchased from Vigorous Biotechnology. The transfection reagent-Hieff Trans™ Liposomal and ECL western blotting detection reagent were from Yeasen Biotechnology. The cell culture mediums were purchased from Invitrogen. All other chemicals were products of analytical grade. The plant-sourced compounds dissolved in DMSO were purchased from Target Molecule Corp.

Cell culture and treatments

The HeLa and HEK 293A cells obtained from ATCC were cultured in the Dulbecco's modified Eagle's medium (Gibco) supplemented with 10% fetal bovine serum (Gibco) and 1% penicillin/streptomycin in a 5% CO_2-humidified incubator at 37 °C. Prior to stress treatment, the cells for immunofluorescence analysis were seeded on glass coverslips pretreated with TC (Solarbio) and plated at 5×10^5 cells/well in 12-well plates overnight. The cells were moved to fresh medium and cultivated in the presence of one of the plant-derived compounds for 4 h. Then the cells were treated with various stressors for give time intervals.

Plasmid constructs and transfection

The gene encoding TIA-1 was cloned from the cDNA library prepared from the HeLa cells

using standard procedures. The obtained gene was inserted into the pEGFP-C3 vector using the following primers: Forward, 5'-ccgctcgagatggaggacgagatgcccaag-3' and Reverse, 5'-cgcgaattcgtcactgggtttcataccc-3'. The recombinant plasmids were transformed into *E. coli* DH5α cells (Transgene Biotech) and confirmed by DNA sequencing. The endotoxin-free plasmids for cell transfection were obtained using the Plasmid Maxiprep kit (Vigorous), precipitated with ethanol and resuspended in sterile TE. The HeLa cells were transiently transfected with 2 μg/ml plasmids containing GFP-TIA-1 using Hieff Trans^{TM} Liposomal (Yeasen Biotechnology) according to the manufacturer's protocol. After transfection for 4-6 h, the cells were moved to fresh DMEM growth medium and cultivated for 24 h.

Isolation of SGs

The HeLa cells transfected with GFP tag TIA-1 were plated into 10 cm plates and grow to 85% confluency. The cells were harvested and washed for two times with 10 ml pre-cold PBS and lysed 15 min at 4 °C in 500 μL lysis buffer (50 mM Tris HCl, pH 7.4, 100 mM KCl, 2 mM $MgCl_2$, 0.5 mM DTT, 0.5% Nonidet P-40, 50 μg/ml Heparin, 1× EDTA-free protease inhibitor cocktail, 1 mM phenylmethylsulphonyl fluoride). The core fraction of stress granules was obtained by differential centrifugation using the published procedures (Jain et al., 2016). In brief, the lysates were centrifuged at 1000 g for 5 min. The supernatant fraction was collected and further separated by centrifugation at 18000 g for 20 min. Then the pellet fraction was resuspended in 500 μL lysis buffer, followed by centrifugation at 850 g for 2 min and the granule-enriched fraction was collected from the supernatants.

Western blot analysis

The separated stress granule cores were incubated with one of the plant-derived compounds at 37 °C for 4 h. Then the samples were centrifuged at 18000 g for 20 min at 4 °C, and the supernatants were collected as the soluble fraction. The pellet was washed with lysis buffer twice and resuspended in lysis buffer. Each sample was mixed with 1× loading buffer and

boiled for 10 min. Denatured proteins were separated by 12.5% acrylamide gel and transferred to poly-vinylidene fluoride (PVDF) transfer membrane (GE). Membranes were immediately blocked for 2 h in PBST containing 5% skimmed milk powder and probed with anti-GFP (1:1000) overnight at 4 °C. Membranes were then incubated with a horseradish-peroxide-conjugated secondary antibody followed by incubation with an ECL reagent. The signals were detected with an Odyssey infrared imaging System (Li-COR Biosciences, Lincoln, NE) and quantified using the NIH ImageJ program. Relative protein levels were obtained from the ratio of precipitation to supernatant fraction and shown as mean ±S.E calculated from three independent experiments.

Cell viability

The HeLa cells were plated into 96-well plates at a density of 2×10^4 cells/well. After growth for 24 h, the cells were cultivated in the presence of one of the compound from the library for 4 h and then treated by various SG-inducing stressors. The cells were treated by 0.5 mM arsenite or 0.2 μM H_2O_2 for 30 min at 37 °C, 1 μM TG or TM for 2 h at 37°C, or heat shock for 2 h at 43°C. Cell viability was measured using the Cell Counting Kit-8. The treated cells were harvested, washed three times by PBS to remove the residual stressors, moved to fresh culture medium and then cultivated in the presence of 10 μl CCK-8 (DoJinDo, CK04) at 37 °C with 5% CO_2 for 30 min. The absorbance at 450 nm was detected using an EnSpire Workstation.

Cell death assay

The HeLa or HEK 293A cells were plated into 6-well plates. After growth for 24 h, the cells were cultivated in the presence or absence of various concentrations of 80 μM C13 or C85 for 4 h and then treated by various SG-inducing stressors. The treatments of various stressors were the same as those used in cell viability assay. After treatment, the cells were harvested, washed by PBS for three times and resuspended in annexin V binding buffer. The cells were

stained with PI and FITC-labeled annexin V (BD Biosciences) according to the manufacturer's instructions. Cell death was determined by separating the propidium iodide- and/or FITC-positive cells using FITC Annexin V Apoptosis Detection Kit (BD Pharmingen 556547) on a FACSCalibur flow cytometer (BD Biosciences). The ratio of dead cells was calculated from three independent experiments.

Immunofluorescence staining (IF)

The treated or transfected cells were washed for three times using phosphate buffered saline (PBS) and then fixed by 4% buffered paraformaldehyde for 40 min at room temperature. The fixed cells were permeabilized with 0.2% Triton X-100 at room temperature for 10 min, washed by PBS for three times. Then the cells were incubated with the blocking buffer (10% FBS in PBS) at room temperature for 1 h, and probed by the primary antibody goat anti-TIA-1 (1:50) in blocking buffer in the dark overnight at 4 °C. The slides were further incubated with Alexa Fluor dyes coupled with anti-mouse or anti-rabbit IgG antibodies at room temperature for 45 min. Cell nuclei were stained with DAPI Fluoromount-G (Southern Biotech). The slides were stored in the dark until being viewed by confocal microscopy (Carl Zeiss LSM 710, ×63/1.4 NA oil immersion objective). The percentage of cells with SGs was calculated from ten randomly selected viewing fields. Deconvolution-crop image-Quick analysis was performed using a DeltaVision Elite microscope (GE Healthcare, ×60 NA oil immersion objective). The exposure time and excitation intensity were kept consistent for the same set of experiments. Fluorescent imaging was analyzed and processed using the Imaris 8.0 software (Bitplane).

Immunoprecipitation (IP) and mass spectrum (MS) analysis

At transfected with plasmids containing TIA-1 for 24 h, the HeLa cells were harvested and lysed for IP and MS analysis. The IP samples were prepared by incubating Protein G Dynabeads (Life Technologies) with 5 μg GFP antibody or the rabbit IgG control according

to the manufacture's instructions. The cell lysates were incubated with the antibody-bound Dynabeads at 4 °C for 4 h. The beads were then washed for three times using the cell lysis buffer. The bound proteins were eluted by boiling the samples with 1× SDS buffer and the supernatants were used for SDS-PAGE analysis. The target bands in the SDS-PAGE gel were further identified via MS analysis.

References

Jain, S., Wheeler, J.R., Walters, R.W., Agrawal, A., Barsic, A., and Parker, R. (2016). ATPase-Modulated Stress Granules Contain a Diverse Proteome and Substructure. Cell 164, 487-498.
